# Drug Delivery Systems on Leprosy Therapy: Moving Towards Eradication?

**DOI:** 10.3390/pharmaceutics12121202

**Published:** 2020-12-11

**Authors:** Luíse L. Chaves, Yuri Patriota, José L. Soares-Sobrinho, Alexandre C. C. Vieira, Sofia A. Costa Lima, Salette Reis

**Affiliations:** 1 Laboratório Associado para a Química Verde, Rede de Química e Tecnologia, Departamento de Ciências Químicas, Faculdade de Farmácia, Universidade do Porto, 4050-313 Porto, Portugal; alexandre.couto@pq.cnpq.br (A.C.C.V.); slima@ff.up.pt (S.A.C.L.); 2 Núcleo de Controle de Qualidade de Medicamentos e Correlatos, Universidade Federal de Pernambuco, Recife 50740-521, Brazil; yuri.patriota@ufpe.br (Y.P.); jose.ssobrinho@ufpe.br (J.L.S.-S.); 3 Laboratório de Tecnologia dos Medicamentos, Universidade Federal de Pernambuco, Recife 50740-521, Brazil; 4 Cooperativa de Ensino Superior Politécnico e Universitário, Instituto Universitário de Ciências da Saúde, 4585-116 Gandra, Portugal

**Keywords:** clofazimine, dapsone, nanoparticles, *Mycobacterium leprae*, targeting

## Abstract

Leprosy disease remains an important public health issue as it is still endemic in several countries. *Mycobacterium leprae*, the causative agent of leprosy, presents tropism for cells of the reticuloendothelial and peripheral nervous system. Current multidrug therapy consists of clofazimine, dapsone and rifampicin. Despite significant improvements in leprosy treatment, in most programs, successful completion of the therapy is still sub-optimal. Drug resistance has emerged in some countries. This review discusses the status of leprosy disease worldwide, providing information regarding infectious agents, clinical manifestations, diagnosis, actual treatment and future perspectives and strategies on targets for an efficient targeted delivery therapy.

## 1. Introduction

Hansen’s disease or leprosy is a non-fatal old disease caused by a bacterium that affects a significant portion of the world population [1,2]. Nowadays, it is one the principal causes of non-traumatic peripheral neuropathy on a global scale [3]. In spite the advances in the political, social, and economic status of developing countries, leprosy is still endemic in many regions, mainly in South East Asia and the Americas [4].

Worldwide, between 2005 and 2014 the number of new cases (about 200,000) was stable [5], thus, in a decade, leprosy continue to be highly prevalent in many regions. In 2019, 13 countries reported more than a 1000 new cases, representing about 95% of all reported new leprosy cases [5]. Brazil, India and Indonesia, disclosed the higher levels of new cases with more than 10,000 new reports representing about 79% of new leprosy cases on a global scale [5]. New approaches have been presented to overcome the stagnation in leprosy control, going from better political commitment, reduction of patient disabilities, to the inclusion of leprosy patients [5].

### 1.1. Ethiologic Agent, the Mycobacterium leprae

The *Mycobacterium leprae* (*M. leprae*) belongs to the order *Actinomycetales* from the *Mycobacteriaceae* family. This bacterium is an acid-fast obligate intracellular bacillus that appears as pleomorphic rods 1 to 8 µm long and 0.3 µm large [6,7]. *M. leprae* replicates by binary diffusion [6,8], in about 12–13 days at 27 to 30 °C [6]. It is a Gram-positive bacteria but can be differentially stained, appearing acid-fast in the Ziehl-Neelsen stain or in the Fite’s acid fast stain [8]. *M. leprae* cannot be grown in vitro, which can be related to its long doubling time (14 days) [7]. The bacteria has been successfully established in vivo by inoculation into the footpad of female Swiss mice, contributing to the development of new antibiotic therapies and the study of drug resistance [7].

**Figure 1:**
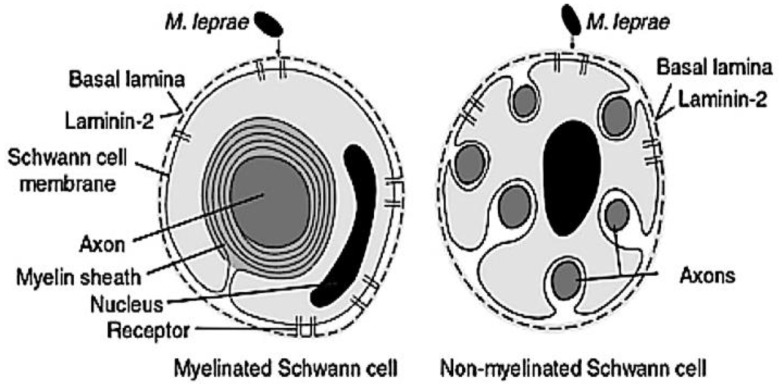
Interaction between *M. leprae* and Schwann cells, mediated by the laminin-α2 present in the basal lamina of myelinated and non-myelinated Schwann-cell–axon units. Reproduced with permission from [8], Elsevier, 2000.

In spite of the high infective rate of *M. leprae*, the disease progression is slow due to the long incubation period of the bacteria [9]. This intracellular pathogen infects preferentially the skin, nasal mucosa and peripheral nerves, exhibiting tropism for reticuloendothelial and peripheral nervous system cells, particularly Schwann cells (SCs) and macrophages [6,10]. Upon entry to the cells, *M. leprae* reorganize in aggregates called globi [9]. *M. leprae* targets SCs by the attachment to laminin-α2 and adhesins and to peripheral nerve surface cell receptors (α-dystroglycan and ErbB2) [1,3]. Upon internalization by the SCs, the bacteria triggers the differentiation into immature cells, suitable for its proliferation [1]. As previously mentioned, *M. leprae* replicates slowly and after its recognition by T cells, initiates a chronic inflammatory reaction [6]. Figure 1 illustrates the interaction between *M. leprae* and laminin-α2 glycoprotein in SCs. Demyelination and disability mediated by loss of axonal conductance are the consequences of *M. leprae* SC infection [3,6,9].

Apart of the SCs, *M. leprae* can successfully survive inside macrophages [11]. It is well-known that mycobacteria, in general, have developed the ability to block the fusion between maturated phagosome with the lysosome in macrophages, which is essential for pathogen destruction, antigen processing and presentation for effective recognition by the adaptive immune system [10]. It has been demonstrated that *M. leprae* uses a mechanism similar to other mycobacteria to avoid this fusion in which a host protein, namely tryptophan aspartate-containing coat protein (TACO, also known as CORO1A or coronin-1), lead the phagosome to escape immune system detection [10,11]. Therefore, CORO1A has been considered an essential host protein that allows the intracellular survival of mycobacteria [10,11].

Likewise, lipid metabolism has been proven to have a crucial role in the survival of *M. leprae* bacilli in the hostile intracellular environment of macrophage or SCs [10]. In fact, skin lesions of severe clinical disease manifestations are characterized by heavily containing infected macrophages (also known as Virchow cells) [12]. These cells present a typically “foamy” appearance, which is in part derived from a classical lipid-droplet marker, called adipose differentiation-related protein, highly positive for infected dermal lesions cells [12,13]. Observation of these cells shows that *M. leprae* resides and replicates within enlarged host-derived lipid accumulation in phagosomes, confirming the importance of lipid metabolism during infection [13,14].

*M. leprae* also suppresses lipid degradation through inhibition of specific lipase expression, resulting in lipid accumulation in the infected macrophages [13,14]. Among these lipids, the cholesterol has been shown to be an essential carbon source for the lipid biosynthesis of *M. leprae* as well as in other mycobacteria. A recent study has demonstrated that cholesterol accumulates in infected macrophages by *M. leprae*, suggesting that the bacilli may be able to dysregulate host cell cholesterol homeostasis by increasing the uptake of native LDL-cholesterol [13].

### 1.2. Classification of Leprosy

Clinical prognosis is the basis for leprosy classification and it helps to distinguish the cases with higher infectiveness [15]. Pathophysiological data is also gathered by the clinical determination of leprosy subtype [15]. The most applied system of classification was established in the 1960s by Ridley and Jopling. A detailed system based on the histology observation of the disease defines five types, from the least to the most severe: tuberculoid (TT), borderline tuberculoid (BT), borderline borderline (BB), borderline lepromatous (BL) and lepromatous (LL) [1]. Between lepromatous and tuberculoid leprosy lies the borderline leprosy, an intermediate manifestation. This leprosy type can be characterized in BT, BB and BL according to patients with clinical, immunological, bacteriological or histological outcomes [15].

In the tuberculoid leprosy few or single small lesions with well-defined and elevated borders (papules and plaques) are found, which may be hyposensitive or anesthetic, and sometimes can be infiltrated [2,7]. Within the damaged tissue, rare acid-fast bacilli are present [8], and when untreated, form copper colored papules or nodules known as leproma. Around the lesion often occurs nerve damage also associated with sensory and/or motor impairment [7].

The transition between TT and LL cases, designated by borderline leprosy depending on the number of clinical signs [2,7]. Usually, asymmetrical and hypoesthetic lesions with peripheral macules or infiltration of the skin are observed [7]. Variability of the lesions is common and the histopathological observation of skin smears may be negative or positive [2]. The BB type is defined by the presence of several non-anesthetic annular lesions with typical infiltrated plaques having an apparent normal skin in the center and a well-defined inner edge [2,7]. BL is characterized by the disseminated presence and symmetric lesions, bilateral and non-anesthetic lepromas and annular lesions [2,7]. The elevated number of the lesion is due to the reduced immunologic resistance [2].

A strong inability for an immunologic reaction against *M. leprae* results in lepromatous leprosy. Patients skin, in particular in the face, earlobes, fingers and toes, present a high number of bilateral and symmetrical, not anesthetic leproma (20 to 100) [6,7]. A large number of bacilli can be found in the dermis [8]. Leprosy manifestations may also occur in the eyes, nose, bones, testis, spleen, liver and adrenals, leading to systemic repercussions and more severe disability [2,6].

In 1982, the WHO recommended a simple therapeutical scheme among the several leprosy types. A new classification for leprosy emerged based on visible symptoms and in the presence or absence of bacilli in slit-skin smears from cooler regions of the body where bacilli proliferate [1,16]. Initially, the treatments schemes were assigned on the basis of the Ridley-Jopling classification, categorizing TT and BT cases of leprosy as being paucibacillary (PB), while BB, BL and LL types were considered multibacillary (MB). In a paucibacillary type, patients present 1 to 5 skin patches and no apparent bacilli in slit-skin smears, while multibacillary condition is defined for more than 5 skin patches and bacilli visible by a microscopic analyses of skin smears. When is not possible to perform slit-skin smears, the criterion for diagnosis is the number of visible lesions [1,16].

**Figure 2:**
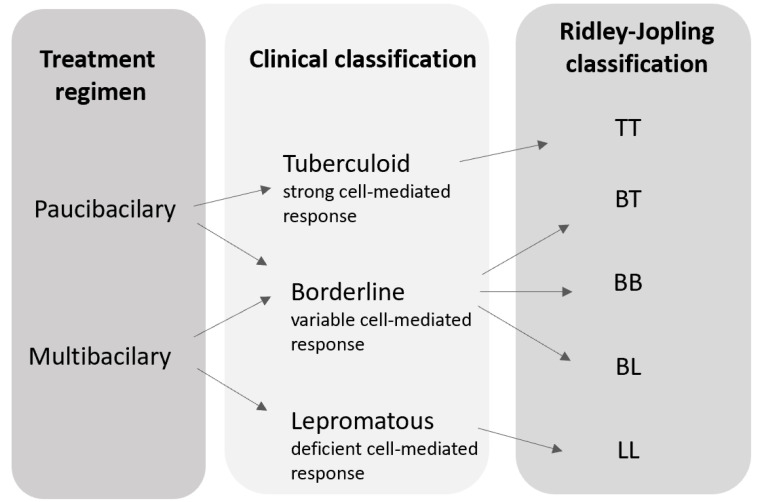
Schematic illustration of leprosy classification.

Leprosy classifications based on the immune response, clinical manifestation and treatment are illustrated in Figure 2.

WHO also implemented another categorization to assess the efficacy of the public health program according to the disability-grading system. This classification has been used as an epidemiological indicator where disability can be defined as any change that may compromise the proper functioning of the body. Disability may be at biological, personal (psychological aspects), environmental or societal (context and participation) levels, experienced by an individual affected by a disease or a special health condition [17,18]. This classification establishes Grade 0 as no impairment, Grade 1 as loss of sensation in the hand, eyes or foot and Grade 2 as visible impairment [17,18].

### 1.3. Diagnosis of Leprosy

The determination of the differential diagnosis demands high levels of expertise and experience in the analysis of the clinical manifestations [19]. Adequate classification of the disease is a crucial step in the success of control programs and treatment scheme standardization [20]. Currently, the diagnosis of leprosy relies on detailed clinical manifestations upon physical examination, and skin biopsy and/or a smear [6,19].

For WHO, the diagnosis of leprosy is based on the presence of cardinal signs, when an individual who has not completed a cycle of treatment has one or more signs: hypopigmented (or erythematous) anesthetic skin lesion; or thickened peripheral nerve or positive skin smear or bacilli observed in a biopsy [6,16].

The first diagnosis assessment of leprosy relies on the patient’s medical history, especially if the individual is coming from, or has recently visited, an endemic country. Second, hypoesthesia skin lesions are indicative of leprosy since there is no other dermatological state associated with sensory disorders [21]. The clinical diagnosis should be confirmed with microbiological and pathological analyses. A microbiological diagnosis is made with samples of skin lesions (smear tests and biopsies). In the microscopic analyses, the samples are stained with the technique Ziehl-Neelsen and the bacterial intensity (BI) determined, which gives information about the gravity of infection and the therapeutic outcome, and is determined after assessing 100 visual fields [22].

Molecular techniques are also applied to complement leprosy diagnosis through a rapid molecular assay using a real-time polymerase chain reaction (RT-PCR) to identify and quantify *M. leprae* DNA in tissue samples [6]. RT-PCR sensitivity is almost 100% for patients presenting positive BI, however, it drops significantly in patients with negative BI [23].

Despite the consolidation of diagnostic and treatment guidelines, the immune responses observed during leprosy are occasionally used to complement clinical diagnosis [24]. Several groups have searched for specific antigens able to detect *M. leprae* infection and confirm the diagnosis, namely the phenolic glycolipid-I (PGL-I) [25] and the leprosy IDRI diagnostic-1 (LID-1) [26], ND-O–LID [27] and antigen 85B (Ag85B; ML2028) [28].

Although advances have been made in the search for novel antigens to detect leprosy, the reactivity of these antigens is high only in MB patients. Strategies such as biotin–streptavidin signal amplification [29] and alternative techniques to exhibit the antigen more efficiently to the patient’s antibodies are under investigation and could overcome these limitations [30].

## 2. Leprosy Treatment Approaches

### 2.1. Conventional Treatment

The past century has witnessed a significant evolution in the leprosy therapy. From the initial strategies based on potassium iodide, arsenic, antimony, copper, sera, vaccines, aniline dyes, thymol, strychnine, baths, X-rays, radium and electrical current [6] to the chaulmoogra oil, a hallmark of therapy in the early 20th century [6]. Promin was the first sulfone drug included to treat leprosy, in the 1940s [31,32]. Then, dapsone monotherapy was implemented with bacilli extinction completed in 3 to 6 months, and complete clinical regression generally occurred within 2 to 3 years [1,33]. However, patients had to maintain the long therapy to prevent the development of resistance [1]. To address this important issue, the WHO, in 1982, introduced a multidrug therapeutic scheme (MDT) with clofazimine (CLZ), dapsone (DAP) and rifampicin, as first line drugs. Patients with PB leprosy, were prescribed a monthly dosage of rifampicin under supervision, and a daily dosage of DAP (non-supervised). For MB patients, the scheme included a monthly supervised intake of rifampicin and CLZ, besides the daily administration of DAP and CLZ without supervision [16]. With this MDT strategy, the course of disease in leprosy patients changed and now it contributes to limiting *M. leprae* dissemination [31].

**Figure 3:**
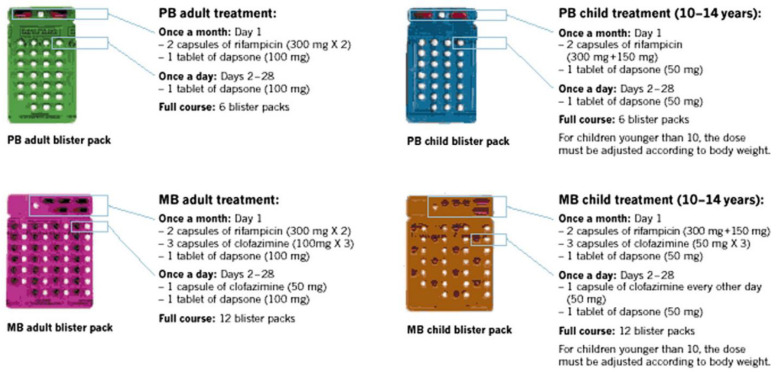
Examples of one month-pack blister freely distributed by WHO for adult and child leprosy treatment. Reproduced with permission from [32], Elsevier, 2015.

For MB leprosy, in all patients presenting LL, BL and BB leprosy, according to the Ridley-Jopling system, the treatment should take up to 12 months, while for PB patients (exhibiting TT, and BT leprosy forms), 6 months are recommended [6,33]. The current time of therapy was revised and initially, the duration of treatment with MDT took 24 months or until smear negativity. This extensive period of treatment led to a significant decrease in patient compliance, resistance and relapse, compared to DAP monotherapy [33]. Since 1995, WHO implemented monthly calendar blister packs, free to all endemic countries, containing the combination of drugs for each type of leprosy [16]. The distribution of the MDT cocktail is made under supervision [31]. Figure 3 represents the current therapy for PB and MB in adult and child patients provided by WHO.

Ofloxacin, minocycline and clarithromycin are alternative agents against *M. leprae* used as second-line drugs in humans but not included in the WHO regimen [1,34]. Yet, a recent study concluded that there is no better treatment scheme than the implemented by the WHO [35]. Efforts to find new combinations of these drugs have been pursued to (i) overcome drug resistance, (ii) shorten therapy duration and (iii) improve the therapeutic efficacy [34].

The fluoroquinolone, ofloxacin (4-fluoroquinolone) is a fluorinated carboxy-quinolone able to act against *M. leprae* through the inhibition of DNA gyrase, DNA replication and transcription [6,8]. It represents an important option in leprosy treatment, particularly for patients with intolerance, resistance or clinical failure to primary therapy [6]. Among the tetracycline group of antibiotics, minocycline is the only one to show significant activity against *M. leprae*, probably due to its lipophilic properties that enhances cell wall penetration. DAP, minocycline and rifampicin combination have an additive activity towards *M. leprae* [36]. Significant antimicrobial activity was described for clarithromycin, a semisynthetic macrolide [8,36]. However, severe gastrointestinal side-effects associated with clarithromycin were sufficient to exclude the use of this drug in the clinical setting [8,36].

### 2.2. Issues and Challenges of Leprosy Treatment

The global incidence of leprosy is still high and long-term complications are often observed in patients [1]. Besides, the MDT confers a microbiological cure but is not sufficient to prevent nerve damage and sequelae associated with leprosy reactions [37]. Note that the statistics commonly do not capture the disability and dysfunction that remain after treatment [1].

Although MDT is the current strategy for leprosy treatment, several drug-resistance cases have been reported worldwide. DAP drug resistance was proved in 1964 with the development of mouse footpad technique for culturing *M. leprae* [23]. Rifampicin and ofloxacin drug resistance were reported in 1976 [38] and 1996 [39], respectively. In addition, a strain of *M. leprae* resistant to both DAP and rifampicin was reported in 1993 [40]. CLZ has been used for leprosy treatment for over four decades; however, only a few cases of drug resistance were reported [41]. Furthermore, resistance to minocycline and other antibiotics effective against *M. leprae* strain such as clarithromycin has not been reported [42].

The appearance of antimicrobial resistance (AMR) in leprosy cannot be disregarded [5]. A uniform multidrug therapy (U-MDT) regimen will allow a shorter period of treatment without supervision for daily drugs (DAP and CLZ) that may lead to AMR [43]. Moreover, knowing the severity of drug resistance, the key for maintaining the effectiveness of MDT is to avoid the spread of drug-resistant strains [42]. Until recently, the mouse footpad model was the only method to investigate *M. leprae* resistance. This method is tedious, not applicable for many samples, time-consuming (6 to 12 months to obtain results), expensive and requires highly skilled technicians. For these reasons, this method is inapplicable for routine use in a surveillance program [44].

The publication of the *M. leprae* genome [45], as well as the understanding of genomic basis of DAP, rifampicin and ofloxacin drug resistance has enabled the identification of a mutation in a drug resistance-determining region (DRDR) through molecular techniques [42]. Due to the lack of information about resistance rates since the introduction of MDT, the WHO Global Leprosy Program established a surveillance laboratory network able to perform molecular detection of resistance in leprosy [46]. The network program in 2008 started with six countries, where *M. leprae* is endemic, and in which 19 countries currently participate. The main objective was monitoring rifampicin resistance, the second and third objectives were to investigate DAP and ofloxacin resistance, respectively.

The first survey on AMR in leprosy from the main endemic countries over a period of seven years was published in 2018 [47]. Rifampicin resistant cases were present in relapses and new cases at a low rate in all continents and WHO regions. They stated that the 8% of resistance rate represents a low threat to MDT treatment but should serve as a baseline for future surveillance studies.

In fact, an AMR surveillance program is present as a central area in the Global Leprosy Strategy 2016–2020: accelerating towards a leprosy-free world [4] and a guide on surveillance of AMR in leprosy was released by WHO [42]. Treatment of leprosy requires further research involving improvement of drug delivery and patient compliance, and effective methods for monitoring drug resistance [37,48]. Efforts to find more antimicrobial agents alone or combined are ongoing to define new future leprosy treatment schemes [33], but so far few novel drugs have shown promising results [6].

### 2.3. Uniform-Multidrug Therapy Regimen

The classification systems established for treatment recommendations has been revealed to be efficient, yet often PB patients are overestimated and MB underestimated, which compromises the adopted regimen [42]. Patient low compliance with long-term treatments in MB-diagnosed patients jeopardizes the therapy success. Among the efforts to uniformize MDT [49], WHO has defined a strategy to eradicate leprosy by 2020 [4]. Based on evidence, the MB treatment regimen was shortened to six months. The inclusion of CLZ in the PB scheme improved the clinical outcome with the disappearance of skin lesions and granulomas [44,49]. So, the uniform treatment regimen for PB- and MB-diagnosed patients evolved into a single regimen for both forms of the disease, the U-MDT, to simplify leprosy control being accessible to primary health care units [4,42]. Ongoing studies will support the revision of current guidelines with a new edition issued by WHO [4].

MB cases present about 61% of the global leprosy patients. The new annually reported cases worldwide are almost all of an MB type [5]. In addition, an important indicator of the management efficiency is the treatment completion rate [5]. Adjustments on treatment duration and therapeutic agents in the U-MDT aim to improve a global approach to reducing disease burden. Proper administration of daily drugs (CLZ and DAP) and patient compliance in MB cases is essential for the efficacy of the treatment.

**Figure 4:**
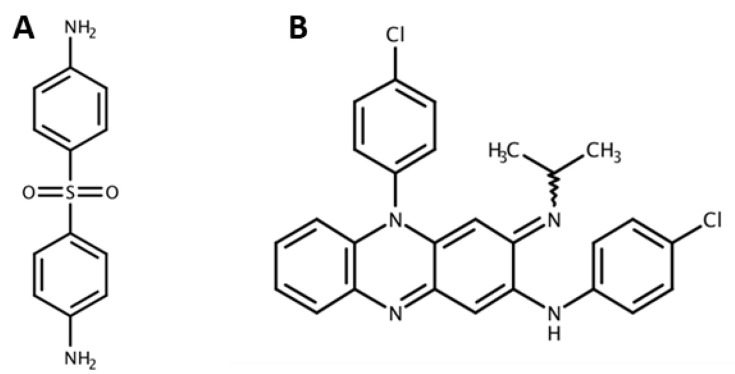
Chemical structures of (**A**) dapsone and (**B**) clofazimine.

The keystone of leprosy therapy is DAP (4,4′-diamino-diphenylsulfone) a sulfone drug, synthetized in 1908 [21,50]. At first, DAP antibacterial activity was not observed [50], only in 1945 was it adopted in the leprosy treatment as monotherapy. However, resistance estimated at 2–10%, has become a problem [6]. DAP is an aromatic amine, with a sulfur atom linking two carbon atoms in its structure, which is crucial for drugs pharmacological activity and toxicity (Figure 4A) [51]. This sulfone is a very weak Lewis base with no readily dissociable hydrogen ion [51] and low water solubility (0.16 mg mL^−1^) being classified as a class II drug (poor solubility and high permeability) according the Biopharmaceutics Classification System [52].

DAP acts as an antimicrobial and anti-inflammatory agent feature common in non-steroidal anti-inflammatory drugs [53]. The antibacterial activity is due to the competitive inhibition of dihydrofolate synthetase, an enzyme involved in the folate biosynthesis pathway in *M. leprae* [6,53,54,55]. Upon oral intake, DAP is absorbed into the gastrointestinal tract (GIT) exhibiting a bioavailability of ca. 80%. Pharmacokinetic parameters show a half-life time between 24 to 30 h, and a serum peak concentration within 2 to 8 h [50,56]. Biodistribution assessment reveals DAP presence throughout the organism, including its ability to cross blood–brain and placenta barriers [56]. After absorption, this sulfone undergoes enterohepatic circulation and suffers liver metabolization resulting in products of N-acetyltransferase and cytochrome P-450 enzymes, namely monoacetyl-dapsone and dapsone hydroxylamine, respectively [50,56]. Excretion occurs in urine as an unchanged drug (ca. 20%) and as water-soluble metabolites (70–85%), and in feces [56].

DAP-associated side effects include digestive problems (e.g., nausea, vomiting and stomatitis), hepatitis, cholestatic jaundice, cutaneous photosensitivity reactions and psychosis [57]. Other adverse effects designated as sulfone syndrome occur due to DAP metabolites (hydroxylamine and other hydroxylated metabolites) and usually involve fever, malaise, jaundice, exfoliative dermatitis or morbilliform rash, hepatic dysfunction, lymphadenopathy, methemoglobinemia, hemolysis, agranulocytosis and hemolytic anemia [32,50,57].

Monotherapy of leprosy has led to an increase in DAP-resistant cases, in the 1960s and 1970s [54], and to overcome this situation other antibacterial agents (CLZ and rifampicin) were included in the leprosy therapy [34]. Resistance may occur upon a mutation in the *M. leprae* folp gene that encodes the dihydropteroate synthase (DHPS) enzyme, which is involved in folate synthesis [6,55].

Clofazimine was initially synthesized as an anti-tuberculosis drug in 1954 [58]. Later, in 1969, it was introduced in the treatment of leprosy [59]. This rhimophenazine acts as an antimicrobial and anti-inflammatory agent, which are both important properties for an efficient leprosy treatment [60]. Key features of this molecule include the phenazine nucleus with an alkylimino and phenyl substituent, which is essential for antimicrobial activity (Figure 4B) [60].

Several studies have been focused on unveiling CLZ mechanism of action. The antimicrobial activity may be associated with the disruption of membrane structure and function [60] or with the increase of phospholipase A2 activity and consequently the release of enzymatic hydrolysis products toxic to *M. leprae* [61]. Evidence has revealed clusters of CLZ and respiratory modulators, suggesting that it may affect electron transport and thus inhibit bacterial cell growth [59].

CLZ is a basic drug of deep red to orange color with three amine groups, protonated and charged at acidic pH and physiological conditions. The color and solubility varies with pH environment, leading often to drug precipitation in vivo, along the GIT [62,63]. CLZ physicochemical properties are governed by very low solubility in water and high lipophilicity (log P > 7), conditions prone to its bioaccumulation as intracellular biocrystals [64]. Oral absorption in humans varies between 45% to 60%, according to the presence or absence of food along with the drug intake. Pharmacokinetics parameters reveal a peak plasma concentration after 8 to 12 h [60]. Upon absorption, CLZ accumulates in lipid-rich tissues, at the reticuloendothelial system and in breasts, liver and intestines [65]. Research reports the ability of CLZ to form complexes with intracellular membranes, precipitating as crystal aggregates [63,64]. Renal and liver excretion is slow, given the drug’s high lipophilicity, usually with an elimination half-life of 70 days [65,66]. Small amounts of CLZ were found in the placenta and in the brain [60]. The described side effects depend on the dosage and affects the skin, eyes and the GIT. Some are reversible upon cessation of therapy, such as the reddish-brown discoloration of the skin and conjunctiva [31,60]. In the GIT, the adverse effects are mild to moderate (abdominal/epigastric pain, nausea, diarrhea, vomiting, gastrointestinal intolerance), or, less frequently, severe (splenic infarction, bowel obstruction, bleeding) [60]. These CLZ-associated toxic effects may be related to patients’ treatment noncompliance [67].

### 2.4. The role of Drug Delivery Strategies in Leprosy Treatment

MDT has been proven as an efficient therapeutic scheme since 1982, and has been fully accepted worldwide for leprosy treatment, as demonstrated by the decline of global disease prevalence [68]. However, it presents several issues as many adverse effects, and treatment is excessively long leading to a low patient compliance [48]. In addition, along the several years adopting the MDT, *M. leprae* drug-resistance has emerged in some countries [44]. So, there is an increasing need for new therapeutic agents and targets that may help to improve the efficacy of leprosy treatment.

The physicochemical characteristics of DAP and CLZ, in particular the poor-water solubility, hamper their therapeutic potential [69]. The success of MDT depends on DAP and CLZ daily dosages, which often exhibit slow dissolution in the GIT, limiting oral bioavailability [70]. Thus, often changes in the administered dosages are needed to reach the therapeutic range, and consequently may worsen toxic side effects [71]. Strategies based on drug delivery systems address the drawbacks related to poorly water-soluble drugs by application of surfactant and lipids, co-solvents, nanocarriers, cyclodextrins and amorphous solid dispersions, among others [71]. A literature review focused on research published for leprosy therapy with DAP and CLZ in different drug delivery systems is summarized in Table 1 and Table 2, respectively.

The most convenient route for drug administration is the oral route since it assures higher patient compliance in relation to the other routes. Few delivery systems designed for oral delivery of DAP are found in the literature (Table 1). A nanometric-sized drug delivery system such as liposomes, polymeric or lipid nanoparticles, usually consists of at least two substances including the active compound. Solid lipid nanoparticles were functionalized with mannose to target intestinal M-cells as a strategy to increase internalization of the DAP by the infected macrophages [72]. The optimized nanoparticles with 300 nm were stably stored and exhibited a pH-sensitive DAP release profile, with a faster drug release at acidic pH than at a neutral pH. Data evidences a promising nanocarrier for treating leprosy with an innovative approach to target DAP directly to M-cells. Targeted intestinal delivery can be accomplished using pH-sensitive polymeric nanoparticles. The enteric pH-dependent copolymer Eudragit^®^ L100 is soluble at pH 6 and was applied to deliver DAP orally [73]. DAP in vitro release from the Eudragit^®^ L100 nanoparticles exhibited the nanoparticles’ pH sensitivity and the safe sulfone deliver in an intestinal environment. The pH-sensitive polymeric nanoparticles showed increased permeation in intestinal cell models compared to the drug solution, thus demonstrating its potential as a therapeutic delivery system for oral regimen in leprosy cases. Smart pH-sensitive hydrogels can also be used to produce oral formulations. Chaves and colleagues have designed chitosan-based hydrogels for DAP oral delivery [74]. High concentrations of the drug were incorporated in the hydrogels (about 8 mg per 28 mg of formulation) and exhibited a controlled drug release under gastro-intestinal conditions. pH-responsive DAP-containing hydrogels represent a new approach for the oral therapy of leprosy.

Oral bioavailability of poorly soluble drugs can be improved using nanoemulsions that enhance drug solubilization, increasing the interface between the lipophilic droplet and the intestinal lumen aqueous medium [81,82]. Monteiro and co-workers developed nanoemulsions with DAP to compare the permeability of formulations with different compositions, namely surfactants and co-solvents, in intestinal cell cultures. The authors observed an increased dissolution rate of the formulations in biological fluid compared to a vehicle-free drug dispersion [75].

Approaches to improve DAP solubility also include macromolecular systems. Solid dispersions are systems in which the drug is molecularly dispersed in a matrix, which promotes increased interactions between the drug and the aqueous medium, facilitating its solubilization [73]. Polymeric solid dispersions obtained with polyvinylpyrrolidone and DAP using the lyophilization technique, evidenced the presence of the drug in its amorphous state with consequent solubility increase [53]. Cyclodextrins are cyclic oligosaccharides with a hydrophilic exterior and hydrophobic interior [83]. This structural feature allows the application of cyclodextrin as solubilizing agents causing hydrophobic drugs to be encapsulated through the formation of a drug-cyclodextrin inclusion complex [83]. Grebogi and co-workers investigated the interactions between DAP and hydroxypropyl-b-cyclodextrin (HPβCD) and β-cyclodextrins (βCD) in the presence and absence of soluble polymers, aiming to increase drug solubility and consequent bioavailability. It was demonstrated that the most stable inclusion complex was obtained with HPβCD, which provided a greater increase in DAP solubility [84].

Human skin provides a unique delivery pathway for therapeutic and other active agents. The main advantages of skin drug delivery include minimal invasiveness or non-invasiveness of an application and improved drug pharmacokinetics and drug targeting. In transdermal drug delivery systems, ethosomal gels present considerable interest due to their good water-solubility and biocompatibility. DAP was incorporated within ethosomes together with an antibiotic cloxacillin sodium, aiming to deliver these drugs to the targeted site more efficiently than marketed gel preparation of DAP, and also to overcome the problems related with the antibiotics’ oral administration [79]. Ethosomal carriers were produced with ethanol (30–40%), cholesterol and soy lecithin and are incorporated in the gel matrix of carbopol, isopropyl myristate and PEG400. The in vitro permeation study confirmed the uniform and sustained permeation of drugs via ethosomal gel. Thus, ethosomal gel of DAP and cloxacillin sodium represent a treatment option for leprosy without any systemic side effect and could also enhance the recovery rate of the skin barrier.

Solid lipid nanoparticles were designed by Kanwar and co-workers, based in the lactonic sophorolipid coated with nonionic polymeric surfactant Pluronics by the solvent injection method for administration of anti-leprosy drugs, rifampicin and DAP [78]. Despite the high drug release rate, the released DAP remained in the therapeutic concentration window for topical administration. Nanoemulsions were developed by Borges and colleagues for topical application of DAP. The use of permeation enhancers in the formulation promoted an increase in the in vitro permeation profile of DAP in the epidermis, which demonstrated the ability of nanoemulsions to overcome the skin barrier [79].

A theoretical study using boron nitride fullerene with a magnetic cluster able to adsorb DAP describes a nano-vehicle candidate for drug delivery [80]. The polarity of the nanocomposites offers the possibility of improving the condition of solubility and of achieving drug dispersion for biological applications. An in silico investigation about covalent and non-covalent interactions between organic molecules and low-dimensional nanomaterials provide useful information for an efficient design of delivery systems.

Lipid nanoparticles are among the strategies commonly applied to improve poorly water-soluble drugs for oral delivery (Table 2). In particular, solid lipid nanoparticles represent the most promising delivery systems to improve the oral bioavailability of hydrophobic drugs. Chaves and colleagues produced solid lipid nanoparticles based on Precirol ATO 5 and Tween 80 using the hot homogenization-ultrasonication method [84]. In vitro cytotoxicity studies revealed that gastric and intestinal cells tolerate more CLZ when loaded in the SLNs than when free in solution. The optimized nanoparticles represent a promising platform for oral CLZ delivery. Formulation of CLZ loaded into polymeric nanoparticles of poly(lactic-*co*-glycolic acid) (PLGA), through a Plackett–Burman design, exhibited an in vitro slow release profile of CLZ [85]. A sustainable release of CLZ is an essential to overcome its recrystallization at the intestinal lumen and within the cells. In fact, CLZ loaded in the polymeric nanoparticles could permeate Caco-2 monolayers substantially at the end of 8 h. Delivery of CLZ using PLGA nanoparticles decreased drug intrinsic toxicity, with improved intestinal permeation.

**Table 2:** Clofazimine drug delivery systems for leprosy therapy.

Type of Delivery System	Composition	Major Outcome	Ref
**Oral Administration**			
Solid lipid nanoparticles	Precirol ATO 5 Tween 80	gastric and intestinal cells tolerate more CLZ when loaded in the nanoparticles than free in solution	[84]
Polymeric nanoparticles	PLGA	nanoparticles decreased drug intrinsic toxicity, with improved intestinal permeation	[85]
**Transdermal administration**			
Liposomes	Phosphatidylcholine Cholesterol HPMC (matrix to deliver liposomes)	liposomal gels were found to be stable at room temperature for up to 3 months	[86]
**Other applications**			
Solid dispersions	Hypromellose phthalate Hypromellose vinylpyrrolidone-vinyl acetate	elucidation of a possible structural model of the drug–polymer complex	[87]
Solid dispersions	Hypromellose phthalate Hypromellose vinylpyrrolidone-vinyl acetate	contribution to a rational selection of appropriate polymeric carriers	[88]

The entrapment of CLZ in a liposomal delivery system for topical application can protect it from absorption into the blood circulation and increase its residence time within the skin. Thus, it may reduce the long mean period of leprosy treatment, as well as the side effects. Patel and Misra produced liposomes composed with phosphatidyl choline and cholesterol and then incorporated them into hydroxypropylmethylcellulose gels for skin delivery of CLZ [86]. Production was optimized in terms of proportion of the composition and methods (vortexing and sonication). The CLZ liposomal gels were found to be stable at room temperature for up to 3 months.

Strong associations between drug and polymeric carriers are expected to contribute to higher drug loading capacities and better physical stability of amorphous solid dispersions. Nie and colleagues produced several amorphous solid dispersions of CLZ with different polymers (e.g., hypromellose phthalate, hypromellose and vinylpyrrolidone-vinyl acetate) by applying the solvent evaporation method [87]. Physicochemical characterization methods elucidate a possible structural model of the drug–polymer complex, as the protonated CLZ bound to the carboxylate group of hypromellose phthalate as an ion pair. Further investigation of these drug–polymer interactions successfully correlated ssNMR findings with quantum chemistry calculations [88]. The high-resolution structural information on CLZ–hypromellose phthalate complex can be useful for the rational selection of appropriate polymeric carriers. The application mesoporous silica nanoparticles to deliver poorly soluble drugs to the sites of diseases is increasing. Chen and co-workers applied a method with acetophenone, an FDA-approved food additive as the chaperone for CLZ. Acetophenone enabled a high amount of CLZ cargo into the mesoporous silica nanoparticles and also allowed effective drug release when in a biorelevant condition [89]. As described, there are very few studies aiming to deliver CLZ for leprosy disease treatment. Nevertheless, CLZ has been successful in treating incidences of multidrug-resistant tuberculosis and is listed as a WHO-recommended second-line drug [90]. For tuberculosis treatment purposes, CLZ has been studied in alternatively administration routes as pulmonary [91,92,93,94], topical [88,95] or other nano-based DDS, alone or in association with other drugs [96,97].

Although DAP and CLZ have been proven to be effective against *M. leprae*, the current drug therapy is threatening to become obsolete. In a general manner, drugs used to treat infectious diseases commonly present limitations as poor physical, chemical, biological and pharmacokinetic properties, as DAP and CLZ, followed by a high risk of acquiring resistance. Co-delivery of multiple anti-infectious agents in a single nano-based system is beginning to show significant advantages over monotherapy, such as synergism, enhanced anti-microbial activity, broad anti-microbial spectrum, reduced resistance development and improved and cost-effective treatment [98]. Until now, only one work has been reported aiming to co-delivery DAP and CLZ simultaneously, although it was intended for tuberculosis and not for leprosy. In 2017, Li and co-workers aimed to develop PLGA nanoparticles with DAP and CLZ through the emulsion solvent evaporation method. The authors found 73 ± 5% and 69 ± 3% of DAP and CLZ entrapment, respectively, and tested its in vitro cytotoxicity on peritoneal mice macrophages, and in vivo efficacy against *Mycobacterium tuberculosis* was studied in an acute model of tuberculosis infection with Sprague-Dawley rats. The results showed that in vitro therapeutic doses of DAP and CLZ were found to be safe to use following the intravenous route of administration. Co-delivery of DAP and CLZ loaded in PLGA nanoparticles offered an effective means of introducing safe drug delivery systemically with enhanced in vivo efficacy against an H37Rv strain of *Mycobacterium tuberculosis* [98].

Another study performed by Chaves and co-workers evaluated the feasibility of drug nanosystems in combination with oral therapy of MB leprosy. DAP and CLZ were incorporated within two polymeric delivery systems, DAP in Eudragit L100 nanoparticles, while CLZ was loaded in PLGA nanoparticles. The permeation of both systems alone and in association were evaluated across a Caco-2 monolayer. It was observed that DAP and CLZ in the nanosystems per se or in NPs-DAP/NPs-CLZ combination crossed the intestinal barrier and there were no significant differences between the single nanosystems or in combination. Although the obtained results seem promising for future drug association for oral delivery of DAP and CLZ, it cannot be considered a co-delivery as the drugs were tested from a different DDS [99].

Despite the efforts engaged in to increase drug aqueous solubility, illustrated by the several types of delivery systems reported in the literature, there is still a gap regarding the efficiency of these systems in eliminating *M. leprae*. The lack of in vitro and in vivo studies relies on the limitation in cultivation of *M. leprae*. Few animal models exist for the study of *M. leprae* pathogenesis in vivo, largely because the ≥37 °C core temperature of traditional rodent models prevents the survival of the mycobacterium. In vitro models that allowed the analysis of host–pathogen interactions inside granulomas have been reported [100]. Wang and collaborators described an in vitro model of *M. leprae* granuloma. Monocytes-derived macrophages were infected in a 24 well-tissue culture plate with *M. leprae* [101]. Regarding in vivo models, *M. leprae* is propagated for research use in the athymic mouse footpad, even though more recently the models using armadillos have been proposed as they may develop neurological disease and form granulomas in response to *M. leprae* [102,103]. More recently, a zebrafish model was proposed in which *M. leprae* growth was shown to be a facile genetically tractable model for leprosy and revealed the interplay between innate and adaptive immune determinants mediating leprosy granuloma formation and function [104].

Aside from the advances in the development of in vivo/in vivo models, none of the drug delivery systems found in the literature with DAP or CLZ had their efficacy tested. This fact discloses the lack of standardization and the limitations of drug development for leprosy.

### 2.5. Targeting Drug Delivery—A Future Perspective

The use of drug delivery systems loaded with different antibiotic drugs coated with specific ligands of host-cell receptors has been extensively explored for many drug-resistant microorganisms. Biomarkers involved in the physiopathology of the infection by *M. leprae* have been identified and are listed in Table 3 as potential targets for effective delivery of antibiotic agents.

In the case of leprosy disease, *M. leprae* can invade the SCs by an interaction between phenolic glycolipid-I (PGL-I), glycoconjugate present on the *M. leprae* surface and the laminin-binding protein of 21 kDa present on the surface of SCs, laminin-α2 [20]. Once attached, *M. leprae* activates SCs-receptor, dystroglycan (DG), causing early nerve degeneration, a major concern in leprosy [108]. Other important receptors present in the SCs surface are the ErB2 and ErK ½ and both are responsible for the *M. leprae*-induced demyelination by MAP kinase [105]. Therefore, this SCs-receptor could be a potential pharmacological target for new antileprotic agents. On the other hand, the knowledge of specific receptors of SCs may represent a key-point for targeted therapies using different drug delivery platforms.

A remarkable aspect of *M. leprae* infection is lipid homeostasis and studies have indicated that it plays an important role in host–pathogen interactions [108]. Cholesterol is one of the host lipid molecules that accumulate in *M. leprae*-infected host cells [107]. Indeed, *M. leprae* is able to induce an imbalance in the complex host cells homeostatic mechanism that tightly regulates cholesterol levels, leading to the formation of “foamy cells”, seen also in tuberculosis [108]. Moreover, and no less important, this cholesterol accumulation suggests a strategy that guarantees bacterium survival as well as a nutrition source [106]. The cellular uptake of native or modified LDL from circulation is a receptor-mediated endocytosis process via LDL-R and scavenger receptors (SRA-1, SRB-2, LRP-1). In fact, the most used pathway for removal of cholesterol from the circulation is the LDL-R [107]. During infection, *M. leprae* induces an overexpression of the abovementioned receptors, suggesting that the increased uptake of LDL may also respond to the accumulation of cholesterol in the *M. leprae*-infected macrophage cells [107]. It was demonstrated that in these “foamy” appearance cells, adipose differentiation-related protein (ADRP), a classical lipid droplet (LD) receptor, is highly expressed, indicating that the foamy form is derived from the LD accumulation [106]. All these findings support the idea that lipid modulation has pathophysiological consequences for bacterial persistence in infected cells. In turn, this knowledge instigates the search of novel targets for pharmacological drugs that could control *M. leprae* infection by acting either directly or indirectly on the host cell metabolic pathways that are critical for bacterial survival.

Toll-like receptors 2 and 6 (TLR-2 and TLR-6) are essential for *M. leprae*-induced LD biogenesis. Moreover, evidence suggests that LD constitutes intracellular sites for eicosanoid synthesis (e.g., PGE2) [106]. Thus, elevated levels of macrophage-generated PGE2 induced by *M. leprae* could act as down regulating the immune response and bactericidal activity [106]. Therefore, TLR-2 and TLR-6 may play a critical role in leprosy pathogenesis by facilitating bacterial persistence in host cells and they have the potential to become targets for novel therapeutic strategies.

## 3. Conclusions

Unfortunately, leprosy disease is still considered an endemic disease in some regions besides WHO efforts to revert this scenario. The MDT represents the most important strategy for this purpose, albeit there is a lack of search in this area, and new drugs or new platforms for drug delivery are scarce. The recommended treatment is commonly unattended, mainly due to the long duration and adverse effects related to the used drugs. In this context, there is a need for a new and deep investigation concerning leprosy disease, considering the physiopathology of the infectious agent and its survival into host cells, which may support the development of new antileprotic agents specific to infected cells. With these future advances, the design of delivery systems coated with specific ligands towards specific targeting of antileprotic agents will help to improve patient compliance with lower and friendly dosages avoiding severe side-effects and will therefore contribute to improve leprosy therapy towards its elimination.

## Figures and Tables

**Table 1:** Dapsone drug delivery systems for oral and transdermal administration.

Type of Delivery System	Composition	Major Outcome	Ref
**Oral Administration**			
Solid lipid nanoparticles	Cetyl palmitate Stearylamine Tween 80 Mannose	exhibits a pH-sensitive DAP release profile, with a faster drug release at acidic pH than at a neutral pH	[72]
Polymeric nanoparticles	Eudragit^®^ L100 Pluronic^®^ F-68 Polyvinyl alcohol	DAP in vitro release assay from the nanoparticles confirmed the nanoparticles’ pH sensitivity; nanoparticles showed increased DAP permeation in intestinal cell models compared to the drug solution	[73]
Hydrogel	Chitosan Glutaraldehyde Hydroxypropyl methylcellulose	loading of DAP within the pH-responsive interpenetrating polymer networks led to in vitro drug controlled release under gastrointestinal pH conditions	[74]
Nanoemulsions	Isopropyl myristate Span 80 or 20 Tween 80, 40 or 20 Propylene glycol Ethanol	DAP release profiles of the nanoemulsions showed a higher dissolution than free DAP	[75]
Polymeric dispersions	Polyvinylpyrrolidone (PVP K30) Pluronic F68	in vivo dissolution rate of PDs was significantly faster compared to free DAP	[53]
Polymeric dispersions	2-hydroxypropyl-β-cyclodextrin (HP β CD) β -cyclodextrin (CD) PVP K30	complex DAP/HP β CD provided a great increase in DAP solubility	[76]
**Transdermal administration**			
Ethosomes	Cholesterol Soy lecitin Ethanol Carbopol 934/PEG 400 with Cloxacillin Sodium	ethosomes in gel matrix exhibited a stable in vitro permeation study with uniform and sustained permeation of drugs	[77]
Solid lipid nanoparticles	Lactonic sophorolipid Pluronics with Rifampicin	released DAP remained in the therapeutic concentration window	[78]
Nanoemulsions	Isopropyl myristate n-methyl pyrrolidone Tween 80 and Span 20	isopropyl myristate promoted an increase in DAP in vitro epidermal permeation	[79]
**Other applications**			
Nanocomposites	Fullerene Boron nitride fullerene	improves the solubility and achieves drug dispersion for biological applications	[80]

**Table 3:** Receptors/macromolecules that participate in *M. leprae* infection.

Receptors of SC	Expressing Cells	Role	Ref
Laminin-α2	Schwann cells	Cause early nerve degeneration	[20]
Tyrosine kinase receptor (ErB2 and ErK ½)	Schwann cells	Result in demyelination	[105]
Adipose differentiation-related protein (ADRP)	Macrophages	Lipid accumulation	[106]
LDL-R	Macrophages	Native cholesterol uptake	[107]
SRA-1, SRB-2, LRP-1	Macrophages	Modified cholesterol uptake	[107]
